# A lake data set for the Tibetan Plateau from the 1960s, 2005, and 2014

**DOI:** 10.1038/sdata.2016.39

**Published:** 2016-06-21

**Authors:** Wei Wan, Di Long, Yang Hong, Yingzhao Ma, Yuan Yuan, Pengfeng Xiao, Hongtao Duan, Zhongying Han, Xingfa Gu

**Affiliations:** 1 State Key Laboratory of Hydroscience and Engineering, Tsinghua University, Beijing 100084, China; 2 Department of Hydraulic Engineering, Tsinghua University, Beijing 100084, China; 3 School of Geosciences, China University of Petroleum, Qingdao 266580, China; 4 Department of Geographical Information Science, Nanjing University, Nanjing 210023, China; 5 State Key Laboratory of Lake Science and Environment, Nanjing Institute of Geography and Limnology, Chinese Academy of Sciences, Nanjing 210008, China; 6 Institute of Remote Sensing and Digital Earth, Chinese Academy of Sciences, Beijing 100101, China; 7 The Center for National Spaceborne Demonstration, Beijing 100101, China

**Keywords:** Limnology, Climate change, Hydrology

## Abstract

Long-term datasets of number and size of lakes over the Tibetan Plateau (TP) are among the most critical components for better understanding the interactions among the cryosphere, hydrosphere, and atmosphere at regional and global scales. Due to the harsh environment and the scarcity of data over the TP, data accumulation and sharing become more valuable for scientists worldwide to make new discoveries in this region. This paper, for the first time, presents a comprehensive and freely available data set of lakes’ status (name, location, shape, area, perimeter, etc.) over the TP region dating back to the 1960s, including three time series, i.e., the 1960s, 2005, and 2014, derived from ground survey (the 1960s) or high-spatial-resolution satellite images from the China-Brazil Earth Resources Satellite (CBERS) (2005) and China’s newly launched GaoFen-1 (GF-1, which means high-resolution images in Chinese) satellite (2014). The data set could provide scientists with useful information for revealing environmental changes and mechanisms over the TP region.

## Background & Summary

The Tibetan Plateau (TP), known as the core region of the Earth’s third pole^1,2^, has attracted great attention from the hydrology, weather, and climate communities. The state of environmental elements of the TP region, such as glacier^3^, permafrost^4^, snow^5^, river^6^, wetland^7^, and lake^8^, is critical for developing a better understanding of the interactions among the cryosphere, hydrosphere, and atmosphere. Lakes, as essential components of the hydrosphere over the TP, play an important role in regional and global biogeochemical processes^9^. Over the last half-century, great efforts have been made to develop a comprehensive understanding of the status and changes of lakes across the TP in previous studies^10–18^.

The state of lakes in early years was mainly recorded by surveying and mapping or field investigations. Afterwards, remote sensing became a powerful tool for lake monitoring since the 1980s. Algorithms for automatic extraction and mapping of lake water bodies from medium and high resolution satellite images have been widely applied^19,20^. However, the accuracy of water boundary extraction using such automatic algorithms still needs further improvement^21^. For studies that require better accuracy or for national-scale survey, semi-automatic extraction or even manual interpretation and digitization seems more feasible for building databases with strict quality control^22^.

There are several global-scale data sets about lakes’ properties, e.g., the Global Lakes and Wetlands Database (GLWD, 1:1 to 1:3 million resolution, freely available at http://www.wwfus.org/science/data.cfm) created using data from many organizations and individuals^23^, the GLObal WAter BOdies database (GLOWABO) produced using GeoCover data set circa 2000 (ref. 24), and a database of summer lake surface temperatures for 291 lakes globally collected in situ and/or by satellites for the period 1985–2009 (freely available at http://portal.lternet.edu/)^25^. However, for regional-scale studies, data sets with higher resolution and longer time series are required. In particular, since the natural environment over the TP region is relatively harsh, data accumulation and sharing can facilitate scientists worldwide in making new discoveries in this region. Although numerous studies on the state of the TP lakes^13,26^ have been performed, there has not been public data set associated with the status of lakes (name, location, shape, area, perimeter, etc.) across the TP region from the past to the present, especially no open-access data set derived from high-spatial-resolution satellite images.

**Figure 1:**
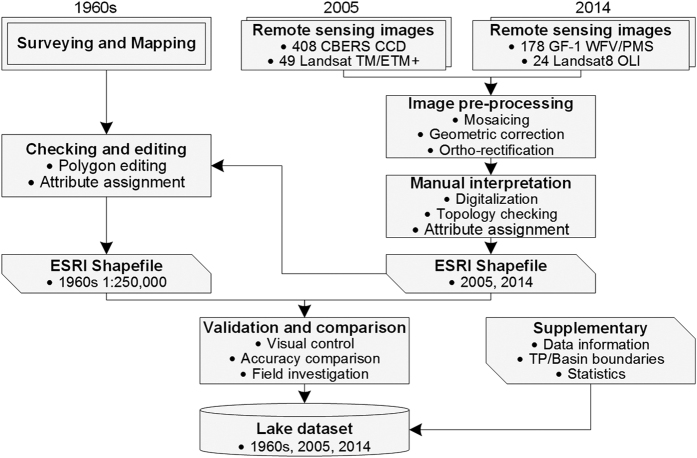
Flowchart for production and validation of the lake data set.

The objective of this study was therefore to produce and share a data set about the state of lakes (area ≥1 km^2^) over the TP. The data set includes three sub-datasets, i.e., the 1960s, 2005, and 2014 time series. The 1960s sub-dataset was produced from a valuable historical record through surveying and mapping, while the 2005 and 2014 sub-datasets were produced mainly using satellite images from the China-Brazil Earth Resources Satellite (CBERS) and China’s newly launched GaoFen-1 (GF-1) satellite, respectively. The 1960s and 2005 sub-datasets originated from the results of the first and second nationwide lake investigations^27,28^, respectively. The 2014 sub-dataset was the first comprehensive evaluation of the GF-1 data for monitoring of TP lakes. Manual interpretation and digitization approaches were applied to ensure the accuracy of the data set. An overview of the production and validation of the data set is shown in Figure 1, and detailed information on methods will be described in the next section. The data set will provide scientists with a useful data source for revealing environmental changes and mechanisms over the TP region. Moreover, the data set could be used to validate automated mapping procedures (e.g., literature^20^ and^29^), to test theoretical hypotheses about lake distributions (e.g., literature^30^), or to contribute to meteorological applications (e.g., literature^31^). For research related to ecology, biogeochemistry, and geomorphology, the value of those even smaller lakes (i.e., area <1 km^2^) is tremendous^32^. Since the data set in this study was produced by manual extraction, this type of lakes was not included. To fill the time gap in the developed data set in this study as well as to extend it in the future, scientists are welcome and appreciated to add smaller lakes, new time series, and new attributes (e.g., water level) into this data set.

## Methods

**Figure 2:**
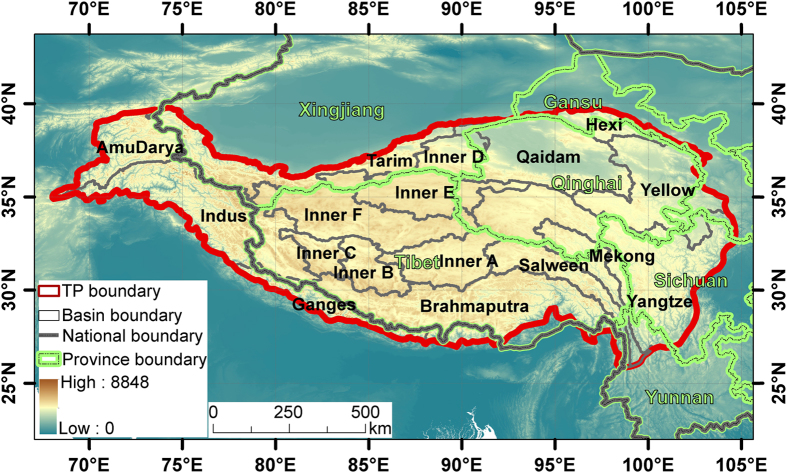
Map of the Tibetan Plateau (TP) region. The region is divided into 12 basins, including 9 exorheic drainage basins (AmuDarya, Brahmaputra, Ganges, Hexi, Indus, Mekong, Salween, Yangtze, and Yellow) and 3 endorheic drainage basins (Tarim, Inner TP and Qaidam). The Inner TP is subdivided into 6 small basins ranging from Inner A to Inner F.

The boundary of the TP in this study is defined as above the elevation of 2,500 m^13^ using the NASA Shuttle Radar Topographic Mission (SRTM) 90 m Digital Elevation Models (DEM) Database v4.1 (Fig. 2). Two Provinces of China, i.e., Tibet and Qinghai, contribute to the major area of the TP (Fig. 2). To make the comparison and analysis convenient, the TP is further divided into 12 basins, including 9 exorheic drainage basins (i.e., AmuDarya, Brahmaputra, Ganges, Hexi, Indus, Mekong, Salween, Yangtze, and Yellow) and 3 endorheic drainage basins (i.e., Inner TP, Tarim, and Qaidam). The Inner TP is subdivided into 6 small basins ranging from Inner A to Inner F. The whole data set includes three sub-datasets: 1960s, 2005, and 2014, and focuses on all the lakes with areas greater than 1 km^2^.

### The 1960s sub-dataset

After the establishment of the People’s Republic of China in 1949, development and utilization of lake resources started to be back on track. New institutions for lake research were established by scientists from governments, universities, and research institudes^27^. In the 1960s, scientists did field surveying and mapping for all the lakes (area more than 10 km^2^) across China, which was part of China’s first nationwide lake investigation and could be traced from literature^27^. All the lakes were coded and published as an industry criterion of China called Code for China Lake Name^33^. A vector database (1:250,000) including the attributes (i.e. location, shape, and area) of the lakes was built. The original version of the 1960s sub-dataset in this paper is a data set extracted from the nationwide 1:250,000 ESRI shapefile format using the TP boundary. Some lakes in the data set have been edited according to the 2005 sub-dataset to be mentioned in the following section, e.g., in the raw 1960 attribute table, one lake may have two or more records directing to the separated parts of this lake, and these parts were merged in this sub-dataset to ensure the uniqueness of the lake attribute. Since the lake surveying was conducted within China, lakes outside the borderline, which were included in the following 2005 and 2014 satellite- based sub-datasets, were not included in the 1960s sub-dataset.

### The 2005 sub-dataset

From 2005–2009, China conducted the second nationwide lake investigation, with the support of the National Key Basic Research Special Foundation of China ‘Lake Water Quality, Water Quantity, and Biological Resources Investigation in China’^28^. Images acquired in 2005–2006 from the CBERS-1 Charge-Coupled Device (CCD) sensor, with a spatial resolution of 19.5 m and temporal resolution of 16 days, were used as the main data source for the investigation. The CBERS is an international technological cooperation program between China and Brazil which developed and operated Earth observation satellites. CBERS-1 was launched in October, 1999, with the CCD camera as its main payload. To obtain intact lake data, images from the Landsat Enhanced Thematic Mapper Plus (ETM+) were used as a supplementary data source during cloudy days for CBERS-1 images. To comprehensively evaluate the state of lakes across the TP, we ensure extraction of information for each lake using two types of images: one was selected in the wet season (i.e., August–October) and the other in the dry season (i.e., April or May). All the CBERS CCD and Landsat ETM+ images were geometrically corrected and geo-rectified to an Albers Equivalent Conical Projection with a Root Mean Square (RMS) uncertainty lower than 30 m. For Qinghai and Tibet Provinces (Fig. 2), images totalling 457 including 408 CBERS CCD images and 49 Landsat ETM+ images were jointly used to extract lake water bodies. The 2005 sub-dataset in this paper consists of two parts: one part is the wet season results of the Qinghai and Tibetan region during the second lake investigation; for lakes outside Qinghai and Tibetan Provinces but inside the TP boundary, we downloaded Landsat ETM+ images (wet season) as supplements to extract the lake boundaries. The two parts were then merged to form the 2005 sub-dataset.

### The 2014 sub-dataset

Images acquired in year 2014 from China’s newly launched GF-1 WFV (Wide Field of View Cameras) sensor were used as the main data source for lake water body extraction. China officially started development of the China High-Resolution Earth Observation System (CHEOS) in May 2010, which was established as one of the major national science and technology projects. The Earth Observation System and Data Center of the China National Space Administration (EOSDC-CNSA) is responsible for organizing the construction of the CHEOS. The space-based CHEOS system was designed to launch 7 satellite series in sequence. GF-1, launched in April 2013, is the first satellite configured with one 2 m panchromatic/8 m multi-spectral (PMS) camera and four 16 m multi-spectral WFV cameras. An 800 km swath-width image can be acquired using the four synchronized-working WFV cameras, which greatly improved the revisit time to 4 days^34^. To match the timing and spatial resolution of the 2005 sub-dataset, the 16 m WFV images during the wet season were used in this study. There are 136 GF-1 images and 11 Landsat8 OLI images used to extract the water bodies. All the GF-1 images were ortho-rectificated before water body extraction. Note that to deal with the problem of missing pixels for Landsat ETM+ SLC-off imagery since 2003 (ref. 35), for both of the 2005 and 2014 sub-datasets, we used multi-temporal images to ensure the accuracy of extraction. Water bodies were firstly extracted in each basin and then merged together to form a whole data set for the TP.

### Water boundary extraction from satellite images

**Figure 3:**
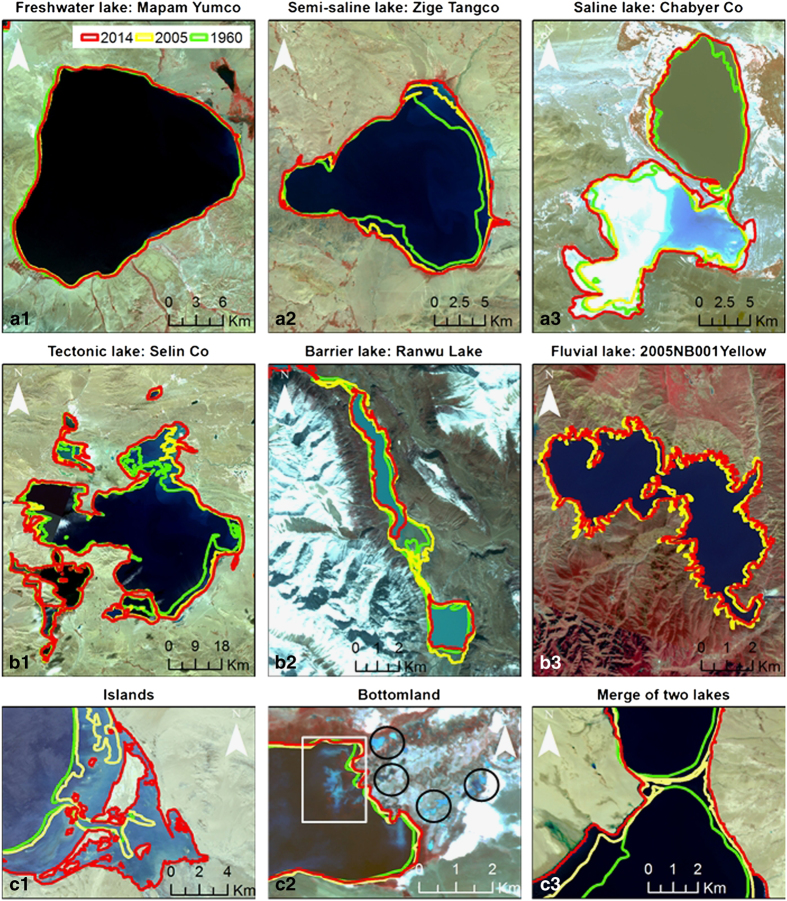
Rules from extracting water bodies of the TP lakes. The first panel: lakes with different water chemical properties (a1-freshwater lake; a2-Semi-saline lake; a3-saline lake); the second panel: lakes with different formation mechanisms (b1-tectonic lake; b2-Barrier lake; b3-fluvial lake); the third panel: performance of islands (c1), bottomland (c2), and the merging of two lakes (c3). The backgrounds are the GF-1 WVF pseudo-color (near-infrared/red/green) composite images.

In order to strictly control the precision of water boundary extraction from satellite images and to provide users with a comprehensive and reliable data set, we chose to manually interpret and extract the water boundaries of the lakes, given possible uncertainties in automatic extracting methods. Note that in this paper, islands inside the lake boundary were not counted to the total area of the lake water surface. Rules for determining water surface boundaries in the TP region are shown in Fig. 3. Green, yellow, and red lines represent the sketched water boundaries of the 1960s, 2005, and 2014, respectively. The three panels (a1–a3, b1–b3, and c1–c3) represent rules for different situations. Details for the rules are explained as follows:

1) *Water body extraction for lakes with different water chemical properties:* Lakes in the TP can be divided into three categories according to their water chemical properties, i.e., freshwater lake, semi-saline lake, and saline lake^27^. Figure 3 a1, a2 and a3 show examples of the appearances for the three categories in GF-1 pseudo-color composite images (near-infrared/red/green) individually. Mapam Yumco (salinity 0.1–0.4 g/l^27^), a freshwater lake in the Indus basin of the southwest TP, shows ultra dark blue (see A1) in the GF-1 image. Zige Tangco, a semi-saline lake (average salinity of 40.7 g/l from field measurements in August, 2010) in the Inner TP basin of the Central Tibet, shows dark blue. The waterlines of both the freshwater lake and semi-saline lake are generally clear in the satellite images. We tracked and drew the waterlines of these lakes while zooming the images into a fixed scale 1:25,000. However, saline lake, like Chabyer Co (salinity 393.5-439.8 g/l^27^) in Figure 3 a3, sometimes has a layer of salt on top of the water surface, which makes it difficult to determine the waterline. For such cases, to ensure the reliability of the results, we checked multiple images in different seasons, and referred to field investigations recorded in the 1960s (ref. 27).

2) *Water body extraction for lakes with different formation mechanisms*: Natural lakes can be formed by various processes. For lakes in the TP, tectonic movement, river erosion, glacial activity, and landslide are the primary drivers for the formation of lakes^27^. Most glacial lakes in the TP are small with areas less than 1 km^2,36^. Therefore, we only focus on describing tectonic lakes, barrier lakes, and fluvial lakes. Figure 3 b1, b2, and b3 show examples of the appearances for the three categories in GF-1 pseudo-color images individually. Selin Co (2300.49 km^2^ in 2014) in B1, a classic tectonic lake lies in Central Tibet, is now larger than Nam Co (2028.50 km^2^ in 2014) and becomes the largest lake in Tibet. The waterline of Selin Co is relatively clear and easy to draw. Ranwu Lake in B2 is a barrier lake formed by the landslide-induced debris flows blocking the river. The water level for barrier lakes is not very high, which makes it light blue shown in the image. In general, the waterline for barrier lakes is pretty clear but it varies with time on occasion. A priori knowledge is important to identify this type of lake. Fluvial lakes are often long and narrow. The lake in Fig. 3 b2 is a new-born lake in 2005 in the source area of the Yellow River. The waterline for this type of lake is highly dentate.

3) *Dealing with specific issues*: Since this data set only includes water bodies of lakes, islands within a lake were removed from the waterline polygon (Fig. 3 c1). Small water bodies in the bottomland of a lake are not included into the total water surface (e.g., the black circles in Fig. 3 c2), except that the water bodies in the bottomland are large enough and connected to the main water body (e.g., the white rectangle in Fig. 3 c2). Figure 3 c3 shows a lake in year 2014 that was merged from two separate lakes in year 2005 or the 1960s. These cases are normal in the northeast of Central Tibet, since most of the lakes have been expending over the past 50 years. In the data set, if a new merged lake was formed from two or more separate lakes, the new lake as a whole was renamed after the larger/largest one of the two/more lakes.

We also determined two specific types of lakes: new-born lakes and dead lakes. For example, if a lake existed in a certain location in the 2005 image while in the 1960s the same location was identified as land or non-lake water body, this lake was defined as a new-born lake. Similarly, if a lake was found in a certain location in the 1960s while in the 2005 image there was no lake in the same location, this lake was defined as a dead lake. Following the above definitions, all the images for years 2005 and 2014 were examined one by one to determine the two types of lakes.

For questionable lakes like ephemeral lakes or salt lakes with salt crusts, we checked and compared their state on both wet-season and dry-season images. If the bottomlands with seasonal-covered water or the salt crusts were located outside the water surface boundaries on both wet/dry-season images, we would not consider them as components of the lake. Otherwise, if they were located inside the water surface boundaries on wet-season images but outside for the dry-season ones, we took the median lines of the water surface boundaries on wet/dry-season images as the lake water boundaries.

## Data Records

The data set is available in three folders. The first folder, ‘Data_Information_File’, contains detailed information on lakes in each sub-dataset, the data collector, image/images used for water body extraction, citations, etc. The second folder, ‘Data_Value_File’, contains two subfolders: ‘shp_1-10’ and ‘shp_10-’, which store the shapefiles of the 1–10 km^2^ and ≥10 km^2^ lakes, respectively. The third folder, ‘Supplement’, contains the boundary files and validation sampling shapefiles. An overall statistical table for numbers and areas in this data set is also included in the ‘Supplement’ folder. The shapefiles of the three sub-datasets can be linked to the lake information via the ID/NAME_CH/NAME_EN columns. The 1960s and 2005 data used in this study have been published in the literature^22,28,37^. The 2014 data have not been published. The data set can be accessed at http://dx.doi.org/10.6084/m9.figshare.3145369 (Data Citation 1).

Table 1 shows data labels and descriptions for the shapefiles in detail. Some lakes may have two or more Chinese or English names. The alias of these lakes are individually recorded after their common names using brackets, e.g., Yazi Lake (Woniu Lake). Lakes that did not have names are recorded as ‘Noname’. The ‘Noname’ lakes are generally small and exist in the ‘shp_1-10’ file folder. An attribute column called ‘IS_NEWBORN’ is used to record whether a lake is a new-born lake or not. Number 0 indicates that the lake is not a new-born lake. Numbers 11, 12, 21, and 22 represent the 1960s–2005 (≥10 km^2^), 1960s–2005 (1–10 km^2^), 2005–2014 (≥10 km^2^), and 2005–2014 (1–10 km^2^) new-born lakes, respectively. All the 1960s–2005 new-born lakes are named by indices and the associated basins, e.g., ‘2005NB001innerF’. In total, for the≥10 km^2^ lakes, four 1960s–2005 new-born lakes were found but no 2005–2014 new-born lake was found. No dead lakes with areas more than 10 km^2^ were found either in 1960s–2005 or 2005–2014. While for the 1-10 km^2^ lakes, twenty 1960s–2005 new-born lakes were found and seven 2005–2014 new-born lakes were found. The ‘NOTES_CH’ and ‘NOTES_EN’ record the notes for specific issues while extracting water bodies for those lakes, e.g., two lakes merged to one single lake due to drastic expansions.

Seven human-exploited salt lakes with area≥10 km^2^, South Huoluxun Lake (96.47° N, 36.44° E), North Huoluxun Lake (95.93° N, 36.91° E), Dabuxun Lake (94.56° N, 36.58° E), East Taiji Nai’er Lake (93.92° N, 37.50° E), West Taiji Nai’er Lake (93.38° N, 37.72° E), Dezongmahai Lake (94.30° N, 38.23° E) and Qarhan Salt Lake (94.45° N, 36.55° E), are not included in this data set, since these lakes are not natural lakes and difficult to extract and analyze their changes. Eleven lakes outside the national boundary of China, including five ≥10 km^2^ lakes and six 1-10 km^2^ lakes, are not included, since the surveyors did not collect the data for these lakes in the 1960s. The five ≥10 km^2^ lakes are Tso moriri (78.32° N, 32.90° E), Karacul Lake (73.41° N, 39.03° E), Sarezskoe Lake (72.78° N, 38.23° E), Yashilkul Lake (72.88° N, 37.77° E), and Shiwa Lake (71.35° N, 37.39° E). The west part of Bangong Co (79.22° N, 33.68° E), a lake stretches across China and Kashmir, is not included as well.

Table 2 shows statistics of the number and area of lakes categorized by basin in the 1960s, 2005, and 2014, respectively. The total number of lakes in 1960s, 2005, and 2014 is 1109, 1197 and 1171, respectively, considering the specific issues mentioned above, e.g., lakes excluded or merged. Overall, during 1960s–2005–2014, continuously increasing trends in the total area are shown in the TP, particularly in the Inner TP basin. More sub-datasets are required to study the detailed information on the change characteristics from 1960s–2005.

## Technical Validation

### Quality control and validation of the dataset

For the 2005 and 2014 sub-datasets, after completing the first round of extracting water body boundaries of all lakes, we had three of the authors of this paper (Wei Wan, Zhongying Han, and Yuan Yuan) cross-check the initial results basin by basin to ensure that there were no missing or erroneous lake. We organized four graduated students to examine the attribute tables for the data set to ensure the validity and integrity. We paid much attention to determining those new-born or dead lakes. Also, we examined ≥10 km^2^ and 1–10 km^2^ shapefiles for the same year together to avoid record repetitions. For the 1960s sub-dataset, we could not examine the fundamental data since the historical surveying and mapping work was unrepeatable. Instead, we examined the 1960s sub-dataset by comparison with the two remote sensing-derived sub-datasets to ensure the consistency of the attributes of lakes, e.g. the ID, name, and located basin.

To achieve robust and quantitative validation of area and perimeter estimates from GF-1 WFV images, we did a two-step comparison. First, we compared the resulting GF-1 WFV-derived values (WFV for short) to the results derived from the Landsat 8 Operational Land Imager (OLI) images (OLI for short), since WFV and OLI have the same level of resolution, i.e. 16 and 30 m. Second, we compared both of the WFV and OLI results with the results derived from the GF-1 PMS images (PMS for short). Here the PMS results were treated as reference data. To achieve a rational sampling number as well as considering the workload, we divided the total number of lakes by area into 6 categories:≥1000 km^2^, 500–1000 km^2^, 100–500 km^2^, 50–100 km^2^, 10–50 km^2^, and 1–10 km^2^. Approx. 5% of the number of each category was selected as samples, i.e. 1, 1, 3, 3, 13, and 33. Names and attributes of the sampled lakes can be found in the ‘Supplement’ folder of the data set. For validation, we collected thirteen Landsat 8 OLI images and fifty-nine GF-1 2 m/8 m PMS images during the wet season in 2014–2015. The raw panchromatic/multispectral images from PMS were firstly ortho-rectificated individually and then processed to create the final 2 m pan-sharpened reference images. The sampled lakes were digitalized out of the OLI and PMS under 1:25000 and 1:2500 scales, respectively. We use two morphometric indices mentioned in Liturature 20, the Shoreline Development Index (SDI) and the thickness index Miller (MI), to describe the morphometry of lakes. Table 3 shows statistics of measured parameters (area, perimeter) and calculated parameters (SDI and MI) for WFV, OLI, and PMS, respectively. In general, the mean, minimum, maximum, and standard deviation for the three data sets are at the same level. The PMS-derived perimeters always showed relatively higher values than the other two, since higher-resolution images could contain more details of lake boundaries.

**Figure 4:**
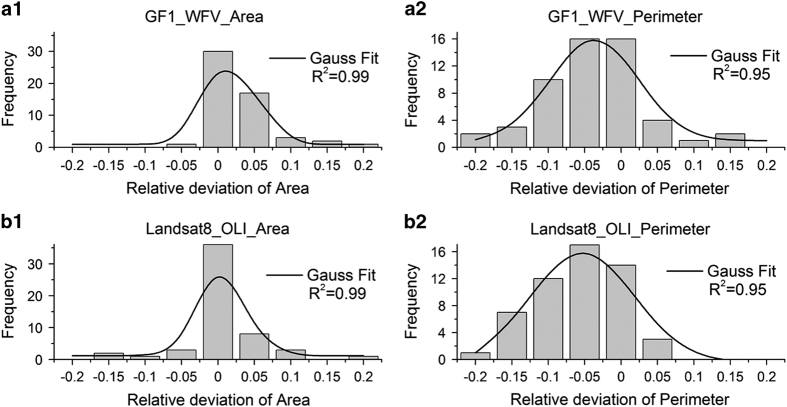
Histograms and Gaussian fits of the relative deviations of GF-1 WFV area (a1) & perimeter (a2), and Landsat 8 OLI area (b1) & perimeter (b2). GF-1 PMS is used as reference data set.

To evaluate the matching of lake boundaries, we calculated relative deviations (RD) in area and perimeter between the WFV, OLI and the respective PMS data sets. For all the sampled lakes, deviation in estimated area for WFV was RD=0.012 (median=0; StDev=0.044), and for OLI was RD=−0.014 (median=−0.012; StDev=0.052). Similarly, deviation in estimated perimeter for WFV was RD=−0.065 (median=−0.074; StDev=0.069), and for OLI was RD=−0.082 (median=−0.07; StDev=0.06). Figure 4 shows histograms and Gaussian fits of the RDs of area and perimeter for the sampled WFV and OLI results. Note that RDs for both area and perimeter distributions reached good R-square values. Based on the error analysis here, the data source and method to create this data set appears to be reliable and robust. It is worth mentioning that since automatic methods are more efficient than manual interpretation, it will be nice to compare these two methods in the future work.

### Comparison with other data set

**Figure 5:**
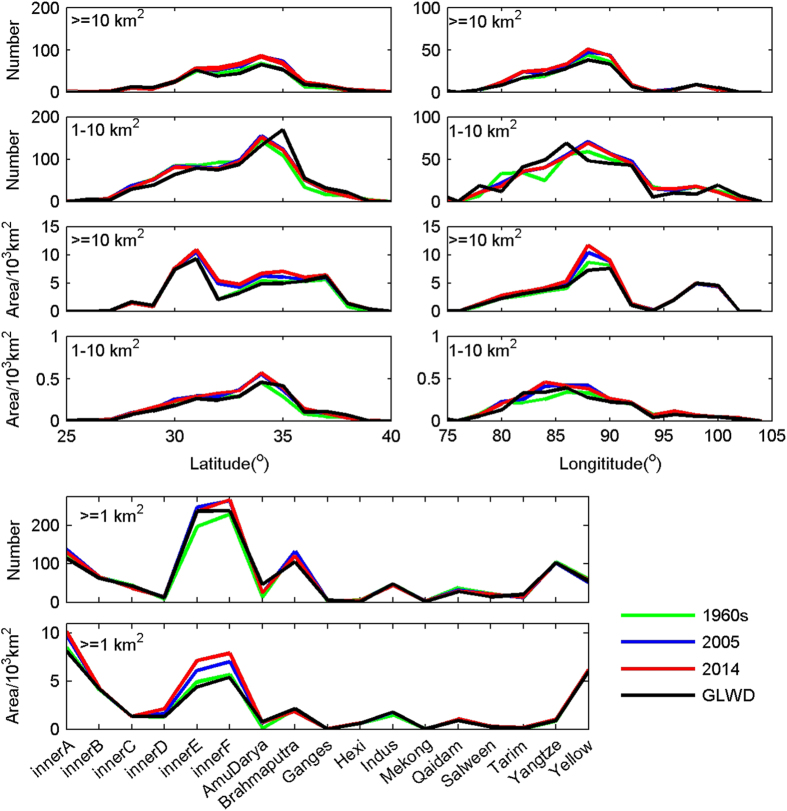
Comparisons of latitudinal, longitudinal and basin-range distributions of numbers and areas of TP lakes for the GLWD data set and the data set produced in this study. Number and area values were aggregated at steps of 1° and 2° for the latitudinal and longitudinal distributions, respectively.

After validating the extraction accuracy of the data set developed in this study, we further compared our data set to another two publicly released data sets, i.e. the global-scale data set GLWD^23^ and a regional-scale data set created by Yao *et al.*^38^. The GLWD was produced using multi-datasources gathered from the 1990s. The level 1 and level 2 of the GLWD data were used for comparison. All the GLWD lakes were firstly extracted using the TP boundary and then regrouped into two categories:≥10 km^2^ and 1–10 km^2^. There are totalling 1131 lakes with an aggregated area of 38,153 km^2^ for the GLWD. This is of the same order of magnitude as that for the data sets in this study. Figure 5 provides an overview of latitudinal, longitudinal and basin-range lake distributions according to different data sets. Number and area values were aggregated at steps of 1° and 2° for the latitudinal and longitudinal distributions, respectively. In general, the data sets in this study showed consistent results as compared to the GLWD. It is reasonable for the inconformity between the two data sets, since they reflect numbers and areas at different time periods. The most striking result of comparing the two data sets, however, is the basic difference in their geolocation for 1–10 km^2^ lakes, e.g., lakes distributed in between 80°–90° E and around 35° N (i.e. northwest of the inner TP basin). For this region, We overlaid the shapefiles of the GLWD and the data sets developed in this study with the 1990s (Landsat 4–5 TM) and the 2014 (GF-1) remote sensing images, and found that some of the GLWD lakes were not shown on the 1990s images, and some small lakes were missing. We checked the lakes in our data sets one-by-one with reference to the GLWD data to ensure that there were no missing lakes. Despite of all the small issues discussed above, we believe that both the developed data sets in this study and the GLWD show good quality. The GLWD data could, to some extent, be a good addition to fill the time gaps in the developed data set in this study.

**Figure 6:**
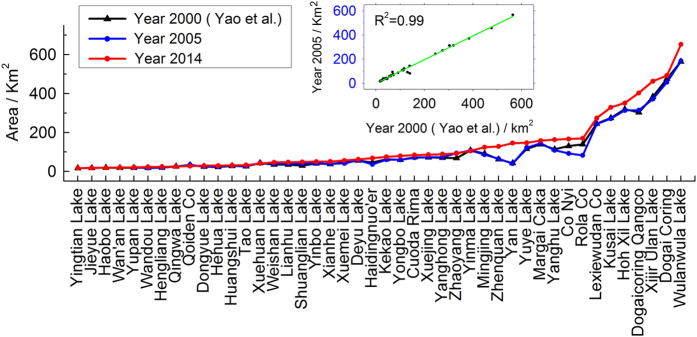
Comparison of the data set in this study and the 2000 (Yao *et al*) results in the Hoh Xil region.

The Yao’s data set was created over the Hoh Xil region using Landsat TM/ETM+ images acquired in 2000. It is noted that the extracting methods and rules are consistent between Yao’s and our data set. There are 44 lakes included in both of the data sets which were selected and used for comparison. Figure 6 shows the area of the target lakes in 2000 (Yao, in black), 2005 (this study, in blue) and 2014 (this study, in red), respectively. Note that for the area of each lake, the 2000 (Yao) data and the 2005 data are basically consistent (*R*^2^=0.99). This is reasonable because the changes in lakes should not be prominent in a 3–5 year period. Some images used for Yao’s results were from years 2001, 2002, and even 2003, making the comparison more convincing. The validation and comparison once again imply that, for lake monitoring, images from various satellite sensors, i.e., CBERS-1 CCD, GF-1 WFV, and Landsat TM/ETM+, can generate consistent and comparable results.

### Assessment of trends in lake changes over the last decades

**Figure 7:**
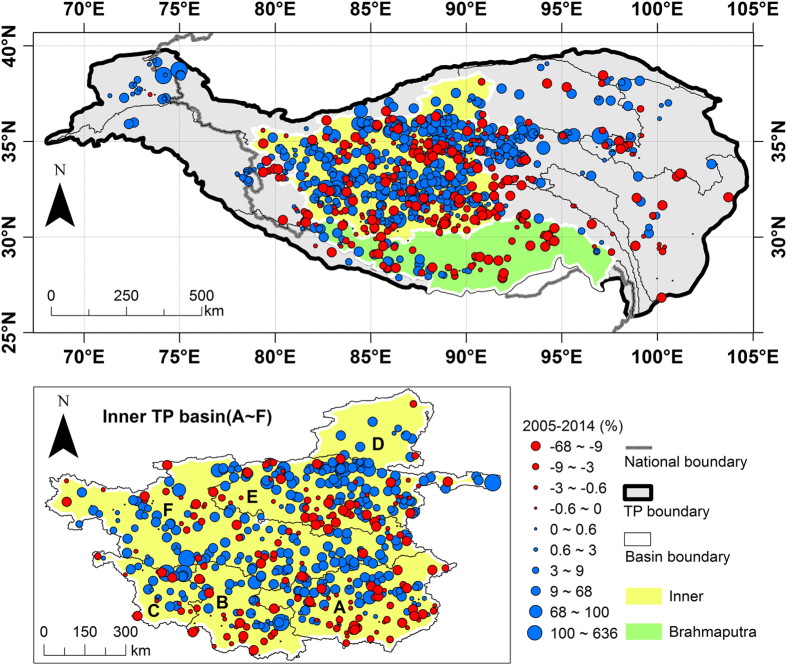
Changing rates of the TP lakes over the last decade (2005–2014). Blue and red solid circles represent positive and negative changing rates, respectively. The Inner TP basin and the Brahmaputra basin are highlighted. The Inner TP basin is zoomed in to show more details.

Figure 7 shows changing rates of lakes in the TP over the last decade (2005–2014). Blue and red solid circles represent increasing and decreasing rates for individual lakes, respectively. Previous studies have revealed that lakes in some TP regions showed consistently expanding or shrinking trends during certain periods. For example, literature^13,39^, using Landsat TM/ETM+ data, suggest that the area of lakes in the inner plateau expanded at a rapid growth rate between the 1990s and 2009/2010 (~27%). Literature^10,18,40^, using ICESat/GLAS altimetry data, suggest that the water level of lakes in the inner plateau showed a significantly increasing trend between 2003 and 2009. Literature^10,13^ reveal that lakes in the Brahmaputra basin showed a decreasing trend in both area and water level. Literature^41^ reveals that the Gravity Recovery and Climate Experiment satellite mission^42^ (GRACE)-derived mass balance rate for the glacialized Himalayan region showed a markedly decreasing trend during 2003–2010. Changing rates of the TP lakes over the last decade (2005–2014) for the Inner TP basin showed in Fig 7 indicate that lakes in this basin have been expanding over recent years (growth rate average 9.88%). The west, middle, and northeast of the basin show a more rapid growth rate (rate averages for Inner C, D, E, and F are 15.63, 15.13, 12.58, and 12.38%, respectively), while the south-eastern part shows a relatively slow growth rate (average rates for Inner A and B are 3.19 and 4.79%, respectively). This demonstrates a consistent and continued trend relative to the findings from the published studies. For changing rates of lakes in the Brahmaputra basin, it is clear that lakes in this basin show a decreasing rate (−2.53%) in recent years. This is highly consistent with the above-mentioned published studies. It is worth mentioning that analyzing the rates of lake change using two time intervals in this study could only obtain a general conclusion. To investigate a particular lake or basin-scale water balance, data acquired at more time intervals and effective automatic methods are required.

## Additional information

**How to cite**: Wan, W. *et al.* A lake data set for the Tibetan Plateau from the 1960s, 2005, and 2014. *Sci. Data* 3:160039 doi: 10.1038/sdata.2016.39 (2016).

## Supplementary Material



## Figures and Tables

**Table 1:** Data labels and descriptions for the shapefiles.

**Data label**	**Description**
ID	Lake code in the industry standard^33^
SHAPE	Feature type of the lake object
NAME_CH	Chinese name of the lake. Some lakes may have alias
NAME_EN	English name of the lake. Some lakes may have alias
LAT_NORTH	North latitude of the geometric centre of the lake polygon in decimal degree
LONG_EAST	East longitude of the geometric centre of the lake polygon in decimal degree
PERIMETER	Perimeter of the lake polygon in kilometre
WATER_S	Area of the lake’s water surface in square kilometre
BASIN	The name of the basin where the lake is located in
IS_NEWBORN	Is the lake a new-born lake comparing to the previous period. 0 represents that the lake is not a new-born lake; 11, 12, 21, and 22 represent the 1960s–2005 (≥10 km^2^), 1960s–2005 (1–10 km^2^), 2005–2014 (≥10 km^2^), and 2005–2014 (1–10 km^2^) new-born lakes, respectively
NOTES_CH	Statements for specific cases in Chinese, e.g. two lakes merged to one single lake due to drastic expansions
NOTES_EN	Statements for specific cases in English, e.g. two lakes merged to one single due to drastic expansions

**Table 2:** Statistics of the number and area of lakes categorized by basin.

**Basin**	**Number**			**Area(km**^**2**^)		
≥10 km^2^	1960s	2005	2014	1960s	2005	2014
1–10 km^2^						
InnerTP						
Inner A	55	62	63	8294.35	9539.48	9949
	72	76	67	203.02	247.3	219.9
Inner B	23	24	25	3962.15	4016.91	4110.62
	42	42	41	146.12	159.86	152.52
Inner C	17	17	17	1219.61	1238.4	1249.12
	27	22	20	97.45	83.14	83.44
Inner D	4	9	9	1278.18	1663.69	2096.83
	4	4	4	14.38	12.73	13.04
Inner E	66	84	82	4552.14	5618.87	6638.09
	130	162	154	347.91	494.63	496.85
Inner F	93	121	128	5233.13	6470.96	7281.65
	136	143	138	425.08	573.34	612.71
Inner TP total	**669**	**766**	**748**	**25773.52**	**30119.31**	**32903.77**
AmuDarya	2	6	6	57.35	587.27	601.72
	11	18	18	33.79	65.6	71.19
Brahmaputra	19	21	18	1772.53	1716.98	1550.77
	113	111	103	272.31	284.29	284.66
Ganges	0	1	1	0	10.8	10.58
	2	3	3	2.68	10.33	10.03
Hexi	1	1	1	599.33	596.39	609.04
	4	3	3	7.99	6.18	6.09
Indus	7	10	11	1373.22	1613.68	1668.54
	41	36	33	113.94	130.04	120.43
Mekong	0	0	0	0	0	0
	3	3	3	16.28	18.12	17.53
Qaidam	17	19	18	994.42	1022.51	1046.15
	20	13	11	47.57	27.62	21.95
Salween	3	3	3	216.33	226.41	230.04
	20	18	18	38.38	44.99	45.95
Tarim	0	3	4	0	45.44	115.98
	11	9	9	40.7	37.41	31.97
Yangtze	13	14	15	592.63	659.94	723.18
	92	88	88	267.93	275.13	295.36
Yellow	17	18	18	5902.73	5863.32	6013.03
	44	33	39	129.87	109.44	149.14
TP total	1109*,†	**1197**†	**1171**†	**38253.5**	**43471.2**	**46527.1**

*Lakes outside the national boundary of China are not included.

^†^Human-exploited salt lakes are not included.

**Table 3:** Measured parameters (area, perimeter) and calculated parameters (morphometric indices, i.e. SDI and MI) statistics of the TP lakes in 2014

	**Area (km**^**2**^)				**Perimeter (km)**				**SDI**				**MI**			
	**Mean**	**Min**	**Max**	**StDev**	**Mean**	**Min**	**Max**	**StDev**	**Mean**	**Min**	**Max**	**StDev**	**Mean**	**Min**	**Max**	**StDev**
≥10 km^2^																
GF1_WFV	186.62	12.57	2028.50	446.64	84.93	15.57	574.54	140.59	1.90	1.21	6.91	1.23	0.41	0.02	0.68	0.19
Landsat8_OLI	185.78	12.00	2029.85	446.67	83.48	15.43	575.65	140.18	1.90	1.22	6.96	1.23	0.41	0.02	0.67	0.19
GF1_PMS	186.81	12.58	2028.79	446.86	88.79	16.07	581.65	145.12	2.01	1.27	6.98	1.23	0.36	0.02	0.62	0.17
1–10 km^2^																
GF1_WFV	4.08	1.38	9.64	2.18	11.63	5.49	22.85	3.92	1.68	1.19	2.39	0.34	0.40	0.18	0.71	0.15
Landsat8_OLI	3.96	1.38	9.24	2.11	11.28	5.46	19.32	3.47	1.67	1.19	2.44	0.33	0.40	0.17	0.71	0.15
GF1_PMS	4.00	1.34	8.53	2.08	12.45	6.33	21.18	3.92	1.83	1.37	2.76	0.39	0.33	0.13	0.53	0.12
Comparisons among GF1-WFV, Landsat8-OLI, and the reference GF1-PMS. The ≥10 km^2^ and 1–10 km^2^ categories are treated separately.																
