# Author Correction: A lake data set for the Tibetan Plateau from the 1960s, 2005, and 2014

**DOI:** 10.1038/s41597-019-0186-3

**Published:** 2019-09-27

**Authors:** Wei Wan, Di Long, Yang Hong, Yingzhao Ma, Yuan Yuan, Pengfeng Xiao, Hongtao Duan, Zhongying Han, Xingfa Gu

**Affiliations:** 1 State Key Laboratory of Hydroscience and Engineering, Tsinghua University, Beijing, 100084 China; 2 Department of Hydraulic Engineering, Tsinghua University, Beijing, 100084 China; 3 School of Geosciences, China University of Petroleum, Qingdao, 266580 China; 4 Department of Geographical Information Science, Nanjing University, Nanjing, 210023 China; 5 State Key Laboratory of Lake Science and Environment, Nanjing Institute of Geography and Limnology, Chinese Academy of Sciences, Nanjing, 210008 China; 6 Institute of Remote Sensing and Digital Earth, Chinese Academy of Sciences, Beijing, 100101 China; 7 The Center for National Spaceborne Demonstration, Beijing, 100101 China; 8 School of Civil Engineering and Environmental Science, University of Oklahoma, Norman, Oklahoma 73019 USA

Correction to: *Scientific Data* 10.1038/sdata.2016.39, published online 21 June 2016

Following publication, it was found that an affiliation of co-author Yang Hong was accidentally omitted from this Data Descriptor. This missing affiliation is given below:

School of Civil Engineering and Environmental Science, University of Oklahoma, Norman, Oklahoma 73019, USA

Accordingly, the email address of Yang Hong has changed to yanghong@ou.edu

